# PAR-CLIP data indicate that Nrd1-Nab3-dependent transcription termination regulates expression of hundreds of protein coding genes in yeast

**DOI:** 10.1186/gb-2014-15-1-r8

**Published:** 2014-01-07

**Authors:** Shaun Webb, Ralph D Hector, Grzegorz Kudla, Sander Granneman

**Affiliations:** 1 Centre for Synthetic and Systems Biology (SynthSys), Mayfield Road, Kings Buildings, Waddington Building office 3.06, Edinburgh EH9 3JD, UK; 2 Wellcome Trust Centre for Cell Biology, University of Edinburgh, Edinburgh EH9 3JR, UK; 3 MRC Human Genetics Unit, University of Edinburgh, Western General Hospital, Crewe Road, Edinburgh EH4 2XU, UK

## Abstract

**Background:**

Nrd1 and Nab3 are essential sequence-specific yeast RNA binding proteins that function as a heterodimer in the processing and degradation of diverse classes of RNAs. These proteins also regulate several mRNA coding genes; however, it remains unclear exactly what percentage of the mRNA component of the transcriptome these proteins control. To address this question, we used the pyCRAC software package developed in our laboratory to analyze CRAC and PAR-CLIP data for Nrd1-Nab3-RNA interactions.

**Results:**

We generated high-resolution maps of Nrd1-Nab3-RNA interactions, from which we have uncovered hundreds of new Nrd1-Nab3 mRNA targets, representing between 20 and 30% of protein-coding transcripts. Although Nrd1 and Nab3 showed a preference for binding near 5′ ends of relatively short transcripts, they bound transcripts throughout coding sequences and 3′ UTRs. Moreover, our data for Nrd1-Nab3 binding to 3′ UTRs was consistent with a role for these proteins in the termination of transcription. Our data also support a tight integration of Nrd1-Nab3 with the nutrient response pathway. Finally, we provide experimental evidence for some of our predictions, using northern blot and RT-PCR assays.

**Conclusions:**

Collectively, our data support the notion that Nrd1 and Nab3 function is tightly integrated with the nutrient response and indicate a role for these proteins in the regulation of many mRNA coding genes. Further, we provide evidence to support the hypothesis that Nrd1-Nab3 represents a failsafe termination mechanism in instances of readthrough transcription.

## Background

RNA binding proteins play crucial roles in the synthesis, processing and degradation of RNA in a cell. To better understand the function of RNA binding proteins, it is important to identify their RNA substrates and the sites of interaction. This helps to better predict their function and lead to the design of more focused functional analyses. Only recently, the development of cross-linking and immunoprecipitation (CLIP) and related techniques has made it possible to identify direct protein-RNA interactions *in vivo* at a very high resolution [1-5]. To isolate direct protein-RNA interactions, cells are UV irradiated to forge covalent bonds between the protein of interest and bound RNAs. The target protein is subsequently affinity purified under stringent conditions, and UV cross-linked RNAs are partially digested, ligated to adapters, RT-PCR amplified and sequenced. CLIP methods are becoming increasingly popular and produce valuable data. The number of papers describing the technique seems to double every year and it is now being applied in a wide range of organisms. The method is also under constant development: the individual-nucleotide resolution CLIP (iCLIP) approach has improved the accuracy of mapping cross-linking sites [2,4], and incorporating photoactivatable nucleotides in RNA can enhance the UV cross-linking efficiency [1]. We have recently developed a stringent affinity-tag-based CLIP protocol (cross-linking and cDNA analysis (CRAC)) that can provide a higher specificity [5], and the tag-based approach is becoming more widely adopted [4,6]. The combination of CLIP with high-throughput sequencing (for example, HITS-CLIP) has markedly increased the sensitivity of the methodology and provided an unparalleled capability to identify protein-RNA interactions transcriptome-wide [3,5,7]. This approach is producing a lot of extremely valuable high-throughput sequencing data. Fortunately, many bioinformatics tools are now becoming available tailored to tackle the large CRAC/CLIP datasets [8-11]. We have recently developed a python package, dubbed pyCRAC, that conveniently combines many popular CLIP/CRAC analysis methods in an easy to use package.

Nrd1 and Nab3 are essential sequence-specific yeast RNA binding proteins that function as a heterodimer in processing and degradation of diverse classes of RNAs [12-19]. Transcription termination of RNA polymerase (Pol) II transcripts generally involves mRNA cleavage and addition of long polyA tails (cleavage and polyadenylation (CPF) pathway), which label the RNA ready for nuclear export (reviewed in [20]). By contrast, transcripts terminated by Nrd1-Nab3 generally contain short polyA tails and are substrates for the nuclear RNA degradation machinery [21,22]. This activity is also important for small nucleolar RNA (snoRNA) maturation and degradation of cryptic unstable transcripts (CUTs) and stable unannotated transcripts (SUTs) [12,23-26]. Nrd1 and Nab3 direct transcription termination of nascent transcripts by interacting with the highly conserved carboxy-terminal domain (CTD) of RNA polymerase II. Because this interaction requires phosphorylation at serine 5 in the CTD, Nrd1 and Nab3 are believed to primarily operate on promoter proximal regions where serine 5 phosphorylation levels are high [27,28].

Recent high-throughput studies have indicated Nrd1 and Nab3 frequently UV cross-link to mRNAs [6,24,29] and thousands of mRNA coding genes harbor Nrd1 and Nab3 binding sequences (see below). However, thus far a relatively small number of mRNAs have been reported to be targeted by Nrd1 and Nab3 [25,30-33]. Indeed, it is not clear exactly what percentage of the mRNA transcriptome these proteins control. To address this question, we reanalyzed CRAC and PAR-CLIP data using the pyCRAC software package. We generated high-resolution maps of Nrd1-Nab3-RNA interactions, focusing on the presence of known RNA binding motifs in the sequencing data. We also confirmed some of our predictions experimentally. Our analyses revealed that Nrd1-Nab3 bound between 20 to 30% of protein-coding transcripts, several hundred of which had binding sites in untranslated regions (UTRs). Although Nrd1 and Nab3 showed a preference for binding near 5′ ends of relatively short transcripts, they bound transcripts throughout coding sequences and 3′ UTRs. Our data suggest that Nrd1-Nab3 can terminate transcription of a long approximately 5 kb transcript by binding 3′ UTRs and we speculate that the fate of many mRNAs is dictated by kinetic competition between Nrd1-Nab3 and the CPF termination pathways. Statistical analyses revealed that Nrd1 and Nab3 targets are significantly enriched for enzymes and permeases involved in nucleotide/amino acid synthesis and uptake, and for proteins involved in mitochondrial organization. Collectively, our data support the notion that Nrd1 and Nab3 function is tightly integrated with the nutrient response [30] and indicate a role for these proteins in the regulation of many mRNA coding genes.

## Results and discussion

### Identification of Nrd1-Nab3 binding sites in PAR-CLIP data

Previous genetic and biochemical studies have identified a number of short Nrd1 and Nab3 RNA binding motifs (UCUU and CUUG in Nab3; UGUA and GUAG in Nrd1) [6,15,16,18,24,29]. Not surprisingly, almost every single mRNA coding gene in the yeast genome contains at least one copy of these motifs and could therefore be Nrd1 and Nab3 targets (see below). To get an impression of how many mRNAs are actually targeted by Nrd1 and Nab3 in yeast, we analyzed data from Nrd1 and Nab3 CLIP/CRAC experiments using the pyCRAC software package [34].

**Figure 1:**
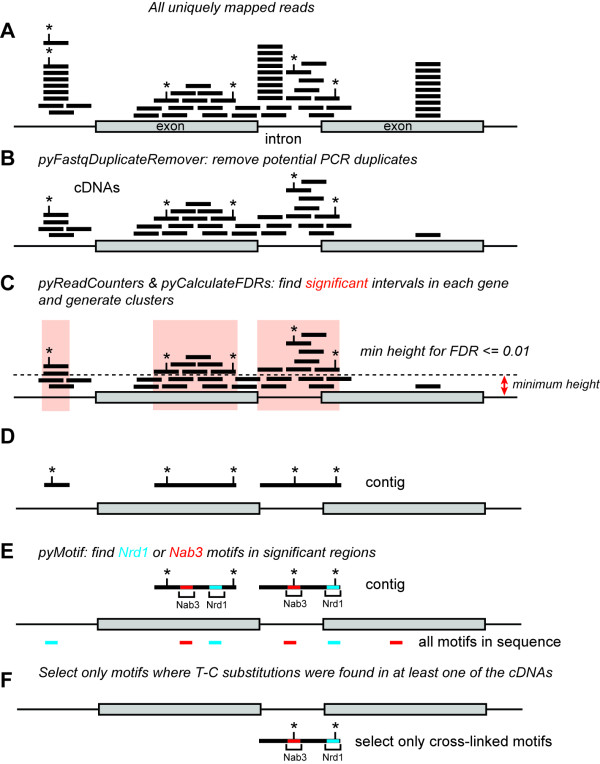
**Schematic overview of the read-processing steps used for our analyses.** Shown is a schematic representation of a gene containing two exons and one intron. Each black line indicates a read and asterisks indicate positions of T-C substitutions. **(A, B)** The first step involved removal of all identical sequences in raw reads by collapsing the data (using pyFastqDuplicateRemover) and aligning the remaining cDNA sequences to the genome. **(C)** pyCalculateFDRs was used to calculate the minimum read coverage height required to obtain an FDR ≤0.01. **(D)** Contigs were generated from significantly enriched regions and T-C mutation frequencies were calculated (using pyCalculateMutationFrequencies). **(E, F)** We then used pyMotif to identify Nrd1-Nab3 motifs in contigs **(E)**, and selected only those motifs where we could find at least one T-C mutation in overlapping reads **(F)**. These are referred to as ‘cross-linked motifs’ throughout the manuscript.

Two high-throughput protein-RNA cross-linking studies on Nrd1 and Nab3 in yeast have recently been described using PAR-CLIP [6,29] and the CRAC method [24]. Both studies produced very similar results and indicated that Nrd1 and Nab3 target RNAs generated by all three RNA polymerases. Here we focus on the PAR-CLIP data, as the number of uniquely mapped reads in these datasets was higher and allowed identification of a greater number of targets (data not shown). Figure 1 provides a schematic overview of how the read data were processed. All identical read sequences were removed and only reads with unique chromosomal mapping positions were considered (Figure 1A,B). Negative control CLIP experiments often do not generate sufficient material for generating high quality cDNA libraries for sequencing. Because no control PAR-CLIP samples were available, we calculated the minimum read coverage (or ‘height’) required to obtain a false discovery rate (FDR) of less than 0.01 for each annotated feature in the genome. Read contigs were generated from those regions with coverage higher than, or equal to, the minimum height (Figure 1C). We reasoned that this approach would reduce noise and sequence representation biases introduced by highly expressed genes. A potential drawback of this approach is that genes with high read coverage (such as tRNAs) are less likely to contain significantly enriched regions, leading to an underestimation of the number of binding sites in these genes.

We next searched for overrepresented sequences in Nrd1 and Nab3 read contigs (Figure 1E). Consistent with recently published work [24,29], previously identified Nrd1-Nab3 motifs were highly over-represented (Table S1 in Additional file 1). Additionally, the recently described AU-rich Nrd1 motifs (UGUAA and UGUAAA) [29,35] were among the top scoring 5- and 6-mers, respectively. Because UV-induced cross-linking sites in PAR-CLIP data are often highlighted by T-C substitutions [1], we reasoned we could obtain higher confidence binding sites by focusing on motif sequences isolated from contigs that contained a T-C substitution in at least one overlapping read (Figure 1D-F). All T-C substitutions in reads were weighted equally and included as mutations in contigs (Figure 1D). Additional file 2 shows that T-C mutations in contigs generated from the Nrd1 PAR-CLIP data were clearly enriched over Nrd1 motifs, confirming that Nrd1 has a strong preference for cross-linking to these sites [6,24,29]. Sequence contigs generated from the Nab3 data sets had high T-C mutation frequencies (Figure S1B in Additional file 2) and only a modest enrichment could be seen downstream of Nab3 motifs. This result is in contrast with recent analyses performed on Nab3 CRAC data, where cross-linking sites were mainly detected within UCUU and CUUG sequences (Figure S1C in Additional file 2) [24]. This discrepancy could be, in part, the result of noise in the Nab3 PAR-CLIP data, as other short sequences were more highly enriched in Nab3 contigs than the previously reported Nab3 binding sites (Table S1 in Additional file 1). To reduce noise, we only selected Nab3 motifs containing T-C substitutions from contigs (Figure 1F), hereafter referred to as ‘cross-linked motifs’. Overall, our motif analyses are in excellent agreement with previously published work.

**Figure 2:**
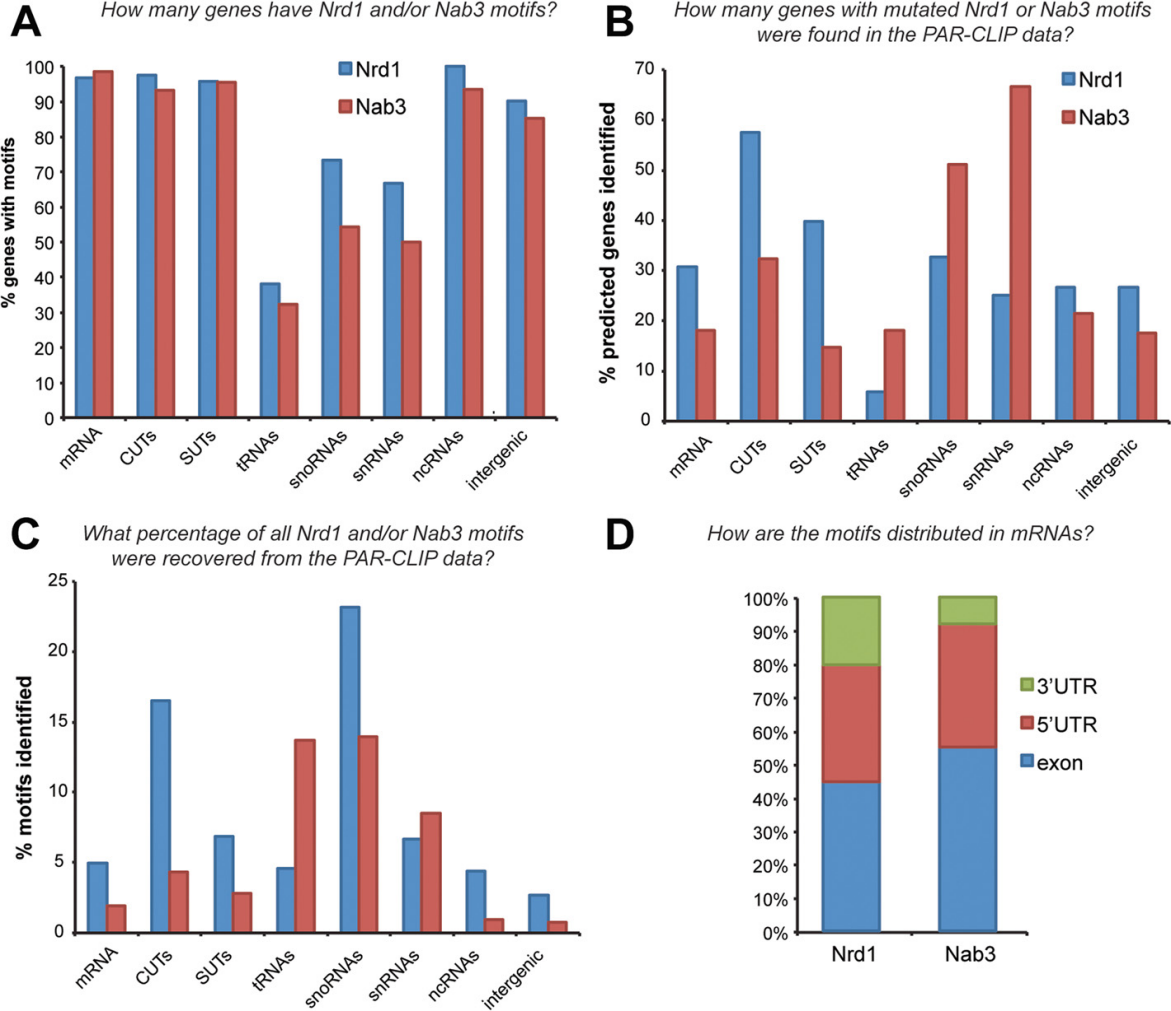
**Comparison of predicted and identified binding sites. (A)** Overview of the percentage (y-axis) of genes in genomic features (x-axis) that contain Nrd1 (blue) or Nab3 (red) motifs in their sequence. **(B)** The percentage of genomic features that contained cross-linked Nrd1 or Nab3 motifs. **(C)** The percentage of all Nrd1 and Nab3 motifs in gene/feature sequences found in the PAR-CLIP data analyses. **(D)** The distribution of cross-linked motifs over UTR and exon sequences. ncRNA, non-coding RNA; snRNA, small nuclear RNA.

### At least a quarter of the mRNAs are Nrd1-Nab3 targets

Figure 2A provides an overview of the percentage of genes in the genome that contain Nrd1 (UGUA, GUAG) and Nab3 (UCUU, CUUG) motifs. The vast majority of motifs were found in protein coding genes and cryptic Pol II transcripts such as CUTs and SUTs. Although generally fewer motifs were present in short non-coding RNA genes (tRNAs, small nuclear RNAs (snRNAs) and snoRNAs; Figure 2A), a high percentage of these motifs contained T-C substitutions in the PAR-CLIP data (Figure 2C). Many Nrd1 and Nab3 motifs are located in snoRNA flanking regions, which were not included in our analyses. Therefore, the number provided here is an underestimation of the total snoRNA targets. Strikingly, the PAR-CLIP analyses showed that Nrd1 and Nab3 cross-linked to 20 to 30% of the approximately 6,300 mRNA transcripts analyzed (Figure 2B), although only a relatively small fraction of all motifs present in the genomic sequence contained T-C substitutions (less than 5%; Figure 2C). Around 50% of the cross-linked motifs mapped to untranslated regions, with a preference for 5′ UTRs (Figure 2D). Consistent with recently published data, our analyses identified the telomerase RNA (*TLC1*) as a Nrd1-Nab3 target [29,36]. Other non-coding RNA targets included the RNase P RNA (*RPR1*), the signal recognition particle RNA (*SCR1*) and *ICR1*. Collectively, our analyses uncovered over a thousand mRNAs that could be regulated by Nrd1 and Nab3.

### Nrd1 and Nab3 preferentially bind to 5′ ends of a subset of mRNA transcripts

**Figure 3:**
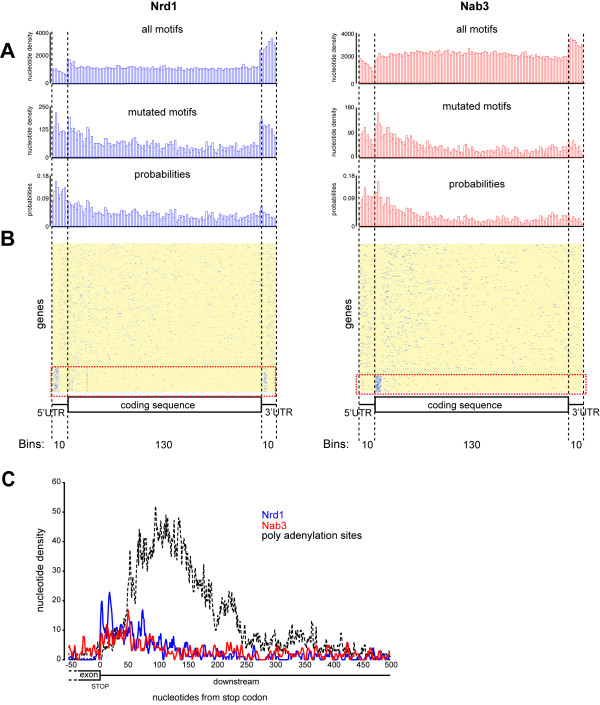
**Distribution of Nrd1 and Nab3 motifs in protein coding regions. (A) Nrd1 and Nab3 preferentially bind near 5′ ends of mRNA transcripts.** Shown are pyBinCollector coverage plots displaying the Nrd1 and Nab3 motif distribution in the exons and UTRs of all non-intronic mRNAs. To normalize the gene lengths the exon sequences were divided in 130 bins and UTRs in 10 bins. Probabilities were calculated by dividing the density values for cross-linked motifs found in the PAR-CLIP data by the density values for all the motifs found in mRNA coding genes. **(B)** Heat map showing the distribution of cross-linked Nrd1 and Nab3 motifs (blue) over individual protein coding genes. pyBinCollector was used to produce a distribution matrix of cross-linked motifs over individual protein coding sequences and the resulting output was k-means clustered using Cluster 3.0. **(C)** Distribution of cross-linked Nrd1 and Nab3 motifs around stop codons and relative to the positions of polyadenylation sites.

**Figure 4:**
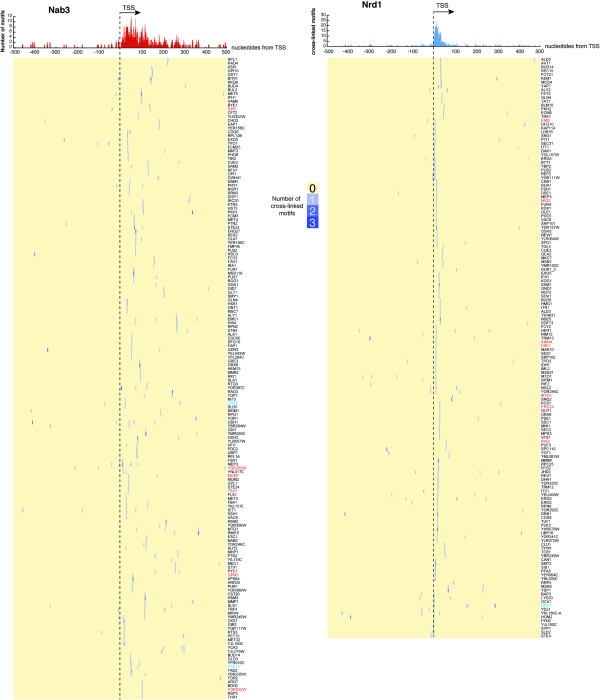
**Distribution of cross-linked Nrd1 and Nab3 motifs around transcription start sites.** The pileup on top of the heat maps indicates the cumulative distribution of cross-linked motifs within a 500-nucleotide window of transcription start sites. The heat map shows the distribution of cross-linked motifs (blue) within individual transcripts. The dashed line indicates the positions of transcription start sites. Red gene names indicate genes where cryptic transcription was detected upstream, whereas cyan colored gene names indicate transcripts previously shown to be regulated by Nrd1-Nab3-dependent transcription termination.

To refine our analyses, we generated genome-wide coverage plots for cross-linked Nrd1 and Nab3 motifs and compared them to the distribution of the motifs present in the genome (Figure 3A). UTR and transcript lengths were normalized by dividing the sequences in an equal number of bins. For each bin we estimated the Nab3/Nrd1 binding probability by dividing the number of cross-linked motifs by the total number of motifs in that bin. To evaluate the quality of the coverage plots, we generated heat maps displaying the distribution of Nrd1 and Nab3 motifs in individual protein coding genes (Figures 3B and 4).

**Figure 5:**
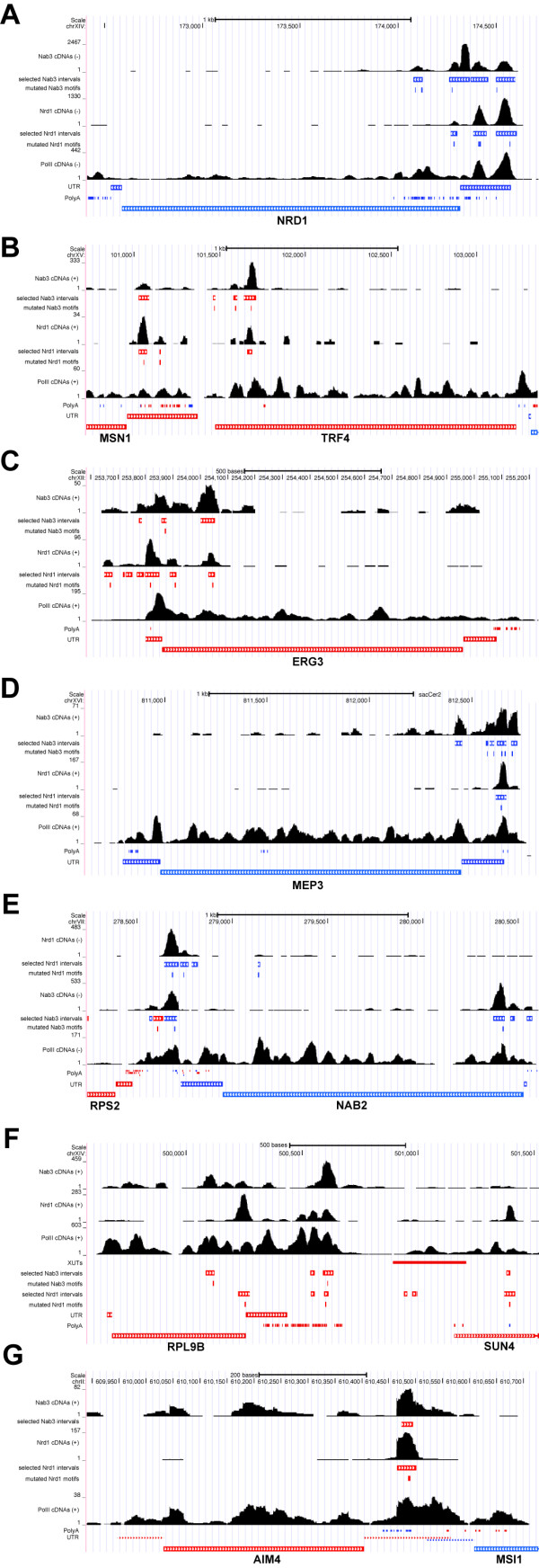
**Nrd1 and Nab3 binding to a selected number of protein-coding transcripts. (A-G)** Shown are UCSC genome browser images for a number of genes predicted to be regulated by Nrd1-Nab3. Coverage of unique cDNAs from Nrd1, Nab3 and Pol II (Rpb2) PAR-CLIP data [6,29] on Watson (+) and Crick (-) strands is shown as black histograms. Locations of cross-linked Nrd1-Nab3 motifs (this work), annotated Xrn1-sensitive unstable transcripts (XUTs), polyadenylation sites and UTRs [22,38-41] are included as rectangles. Genomic features located on the Watson (+) strand are indicated in red, whereas features on the Crick (-) strand are indicated in blue. ‘Selected intervals’ indicate genomic regions with a read coverage FDR ≤0.01. These were used for pyMotif analyses.

Both Nrd1 and Nab3 are co-transcriptionally recruited to the Pol II CTD. Chromatin immunoprecipitation (ChIP) experiments have indicated a preference for Nrd1-Nab3 binding near the 5′ ends of protein coding genes [27,28,37]. Binding of Nrd1 and Nab3 near the 5′ end of transcripts can lead to premature transcription termination and it was proposed that this was a regulatory mechanism for downregulating mRNA levels. Indeed, transcriptome-wide, the probability of finding cross-linked motifs was higher near the 5′ end of protein coding genes (Figure 3A). However, the heat maps in Figure 3B show that the distribution of cross-linked motifs over mRNAs varied considerably, and indicated that a relatively small number of genes mostly contributed to the signal near 5′ ends. K-means clustering of the pyBinCollector data revealed 308 transcripts where cross-linked Nrd1 and/or Nab3 motifs concentrated near 5′ ends (highlighted by a red-dotted line in Figures 3B and 4), primarily downstream of the transcription start site (TSS) (Figure 4). This group included previously described Nrd1-Nab3 targets, such as *PCF11, URA8* and *NRD1* (Figures 4 and 5A) [6,25,29] and therefore may represent a group of genes that are regulated by Nrd1-Nab3-dependent premature transcription termination. Notably, this group also included numerous other genes required for mRNA 3′ end formation as well as genes encoding turnover and export factors (Figures 4 and 5B; *PAP2/TRF4, PTI1, REF2, DHH1*, *NAB2*, *TEX1, PTI1, NOT5*). We speculate that Nrd1 and Nab3 can regulate mRNA metabolism at many levels.

Gene Ontology term analyses on this list of transcripts also revealed a significant enrichment of enzymes with oxidoreductase activity (almost 10%; *P*-value <0.02) and genes involved in cellular transport activities such as nitrogen compounds (8.8%; *P*-value = 0.0069). These included genes involved in ergosterol biosynthesis (Figure 5C; *ERG24*, *ERG3* and *ERG4*), nucleoporins (*KAP114*, *KAP108/SXM1*, *KAP121/PSE1*, *KAP142/MSN5*), several nucleoside and amino acid permeases (*FUR4*, *MEP3*, *MMP1*, *DIP5*, *CAN1*, *FCY2, BAP3*; Figure 5D) and various other transporters (*TPO1, TPO3, TAT1, YCF1*).

Regulation of many genes involved in nucleotide biosynthesis is dictated by nucleotide availability and involves selection of alternative TSSs (*IMD2*, *URA2*, *URA8* and *ADE12*) [42-45]. When nucleotide levels are sufficient, transcription starts at upstream TSSs and the elongating polymerase reads through Nrd1-Nab3 binding sites. When Nrd1-Nab3 bind these transcripts they are targeted for degradation. Indeed, several of the transcripts that originate from alternative TSSs have been annotated as CUTs. For a number of genes we could also detect cross-linked motifs upstream of the TSSs. Interestingly, cryptic transcription (XUTs and/or CUTs) was detected just upstream of *AIM44, CDC47/MCM7, DIP5, ERG24, EMI2, FCY2, FRE1, GPM2, IRA2, MIG2, MYO1, TIR2, TEX1, YOR352W* and *YGR269W*[38,39] (red colored gene names in Figure 4), hinting that these genes could also be regulated via alternative start site selection.

Collectively, these data are consistent with a role for Nrd1 and Nab3 in the nutrient response pathway [30] and we speculate that Nrd1-Nab3-dependent premature termination is a more widely used mechanism for regulating mRNA levels than was previously anticipated [25].

### Nrd1 and Nab3 bind 3′ UTRs of several hundred mRNAs

Nrd1 and Nab3 have been shown to regulate expression of mRNA transcripts by binding 3′ UTRs. It was proposed that in cases where the polymerase fails to terminate at conventional polyadenylation sites, Nrd1 and Nab3 binding to 3′ UTRs could act as a transcription termination ‘fail-safe’ mechanism [32]. From our data we predict that this is likely a widely used mechanism to prevent Pol II from transcribing beyond normal transcription termination sites.

We identified a total of 373 transcripts (approximately 6% of all protein coding genes analyzed) where cross-linked Nrd1 and/or Nab3 motifs mapped to 3′ UTRs (Table S2 in Additional file 1). Two examples are shown in Figure 5B,E. We identified several cross-linked Nrd1 and Nab3 motifs downstream of the *MSN1* and *NAB2* coding sequences. We speculate that these are examples of ‘fail-safe’ termination, where Nrd1 and Nab3 prevent read-through transcription into neighboring genes located on the same (*TRF4*) or opposite strand (*RPS2*). This arrangement of termination sites is reminiscent of the region downstream of *RPL9B* (Figure 5F), where the CPF and Nrd1-Nab3 termination machineries act in competition [33]. Cross-linked Nrd1 motifs also appeared enriched near the 3′ ends of protein coding genes (Figure 5A,B). The Nrd1 G*UAG* and G*UAA* motifs contain stop codons and we found that indeed a fraction of the cross-linked Nrd1 motifs recovered from the PAR-CLIP data overlapped with stop-codons (Figure 5C).

**Figure 6:**
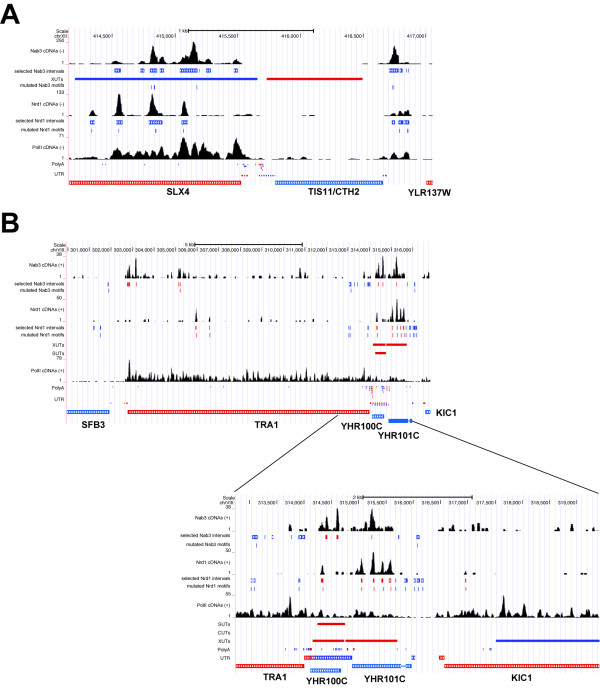
**Nrd1 and Nab3 binding to *CHT2*, *SLX4 *and *TRA1 *transcripts. (A, B)** Coverage of unique cDNAs from Nrd1, Nab3 and Pol II (Rpb2) PAR-CLIP data [6,29] on Watson (+) and Crick (-) strands is shown as black histograms. ‘Selected intervals’ indicates genomic regions with a read coverage FDR ≤0.01 used for pyMotif analyses. Locations of cross-linked Nrd1-Nab3 motifs (this work), annotated XUTs, CUTs, SUTs (if present), polyadenylation sites and UTRs [22,38-41] are included as rectangles. Genomic features located on the Watson (+) strand are indicated in red, whereas features on the Crick strand (-) are indicated in blue.

A role for Nrd1-Nab3-dependent 3′ end processing of mRNA has also been described: the *TIS11/CTH2* mRNA is generated from approximately 1,800-nucleotide, 3′ extended precursors and binding of Nrd1 and Nab3 to 3′ UTRs recruits the exosome that is responsible for trimming the extended RNAs [31]. Our analysis identified 6 cross-linked Nrd1-Nab3 motifs within this 1,800 *CTH2* nucleotide region (Figure 6A) and we could find several other examples of genes with a similar organization of binding sites. One striking example was *TRA1*, a component of the SAGA and NuA4 histone acetyltransferase complex (Figure 6B). Several Nrd1-Nab3 peaks and four cross-linked Nrd1 motifs were identified downstream of the *TRA1* coding sequence. Notably, the downstream regions of *CTH2* and *TRA1* overlap with transcripts annotated as ‘anti-sense regulatory non-coding RNAs’ (Xrn1-sensitive unstable transcripts (XUTs)) [46], raising the question of whether these XUTs are products of read-through transcription.

### Nrd1-Nab3 and mitochondrion organization

The Corden laboratory recently demonstrated a role for Nrd1 in mitochondrial DNA maintenance [30]. An *nrd1-102* temperature-sensitive mutant showed a higher mitochondrial DNA content and was synthetically lethal with an *AIM37* deletion, a gene involved in mitochondrial inheritance [30,47]. Remarkably, a statistically significant fraction of the cross-linked Nrd1 and Nab3 motifs located in 3′ UTRs mapped to genes involved in mitochondrial organization and maintenance (37 genes, *P*-value 0.011). These include those encoding the mitochondrial DNA binding protein (*ILV5*), the nuclear pore associated protein (*AIM4*; Figure 5G), a large number of proteins that localize to the mitochondrial inner membrane (*COX16*, *COX17*, *FCJ1*, *TIM12, TIM14/PAM18*, *TIM54*, *YLH47*, *YTA12*, *CYC2, COA3, OXA1*) and several mitochondrial ribosomal proteins (*NAM9*, *MRP13*, *MRPL3*, *MRPL21*, *MRPL22* and *MRPL38*). Notably, cells lacking *AIM4* show similar defects in mitochondrial biogenesis as an *aim37*Δ strain [47].

Collectively, the data suggest that Nrd1 and Nab3 play an important role in mitochondrial function and development.

### Nab3 is required for fail-safe termination of the convergent HHT1 and IPP1 genes

**Figure 7:**
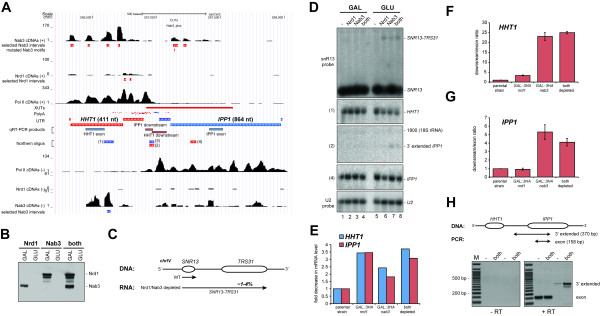
**Nab3 is required to suppress cryptic transcriptional activities. (A)** UCSC genome browser images of the region showing HHT1 and IPP1. ‘Selected intervals’ indicate genomic regions with a read coverage FDR =0.01 used for pyMotif analyses. See the legend to Figure 5 for additional details. Chromosomal positions of RT-PCR products and northern blot probes are also indicated. **(B)** Western blot displaying levels of 3HA-tagged Nrd1 and Nab3 proteins before and after the shift to glucose. Experimental details are provided in the Materials and methods. Proteins were detected using horse radish-conjugated anti-HA antibodies (Santa Cruz). **(C)** Schematic representation of transcripts generated in the SNR13-TRS31 region of yeast chromosome IV (adapted from [13]). About 1 to 4% of the SNR13 transcripts were read-through transcripts in Nab3 and Nrd1 depleted cells, respectively. **(D)** Northern blot analysis of IPP1, HHT1, snR13 and U2 snRNA and 3' extended species. Shown are phosphoimager scans of a blot probed with various oligonucleotides (indicated on the left of each panel). U2 snRNA levels were used as a loading control. **(E)** Depletion of Nrd1 and/or Nab3 results in a reduction of HHT1 and IPP1 mRNA levels. The mRNA levels were quantified using the AIDA software package and normalized to both the levels in the parental strain and the U2 snRNA. **(F, G)**. Quantitative RT-PCR analysis of HHT1 and IPP1 transcription in coding sequences (exon) and downstream regions. Fold change in transcription downstream of these genes was calculated by normalizing the data of the downstream regions to the signals obtained for the exon region. Error bars indicate standard deviations **(H)** Detection of IPP1 read-through transcripts by end-point RT-PCR. The diagram indicates the regions amplified. The position of 3' extended products and exon fragments in the gel are indicated on the right of the gel image.

**Figure 8:**
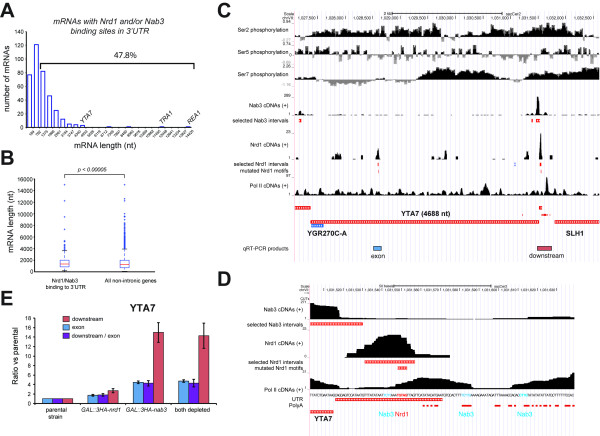
**Nrd1 and Nab3 can terminate transcription of long transcripts by binding to 3′ UTRs. (A, B)** Nrd1 and Nab3 preferentially bind transcripts approximately ≤1 kb. The histogram in **(A)** shows the length distribution (including UTRs) of transcripts bound by Nrd1 and Nab3 in the 3′ UTR. Only transcripts where cross-linked motifs mapped to the 3′ UTR were selected. The bracket indicates the percentage of transcripts longer than 782 nucleotides. The boxplot in **(B)** shows a comparison of the length distribution of the transcripts in **(A)** with the length distribution of all non-intronic protein coding genes in yeast. The *P*-value was calculated using a two-sample Kolmogorov-Smirnov test and indicates the likelihood that the two samples originate from the same continuous distribution. **(C, D)** UCSC genome browser images of *YTA7* region. ‘Selected intervals’ indicate genomic regions with a read coverage FDR ≤0.01 used for pyMotif analyses. The Pol II serine phosphorylation ChIP data were obtained from [37]. See the legend to Figure 5 for more details. Chromosomal positions of RT-PCR products are indicated below the *YTA7* gene. The Nab3 and Nrd1 motifs in the approximately 100 bp region downstream of *YTA7* are indicated in cyan and red, respectively. **(E)**. Quantitative-RT-PCR results for *YTA7* coding sequence (exon) and downstream region. Error bars indicate standard deviations.

To substantiate our results we analyzed expression levels of several genes that we predicted were regulated by Nrd1-Nab3 (Figure 7A). For these analyses we used strains in which the Nrd1 and Nab3 genes were placed under the control of a galactose inducible/glucose repressible promoter (GAL/GLU; Figure 7B), allowing us to deplete these proteins by growing the cells in glucose-containing medium using well established conditions [24]. Transcript levels were analyzed by northern blotting and/or RT-PCR (endpoint and quantitative; Figures 7 and 8). Consistent with previous work [13], northern blot analyses showed that depletion of Nrd1 and/or Nab3 resulted in read-through transcription beyond the *SNR13* gene through the *TSR31* gene (Figure 7C,D). Under the depletion conditions used, between 1% (Nrd1-depleted) and 3.5% (Nab3-depleted) of the *SNR13* RNAs were read-through transcripts (Figure 7C).

The convergent *HHT1* and *IPP1* genes came to our attention because we identified a cross-linked Nab3 motif that mapped to a XUT located directly downstream of the *HHT1* gene (Figure 7A). XUTs can silence expression of neighboring sense genes by modulating their chromatin state [46]; therefore, this XUT could play a role in regulating *IPP1* expression. In addition, substantial Nab3 cross-linking was also observed to anti-sense *HHT1* transcripts (Figure 7A). We predicted that Nab3 was required to suppress multiple cryptic transcriptional activities in this region.

Quantification of the northern data shown in Figure 7D revealed a two- to four-fold reduction in *HHT1* and *IPP1* mRNA levels in the absence of Nrd1 and/or Nab3 (Figure 7E). These results indicate a role for Nrd1 and Nab3 in regulating mRNA levels of these genes.

We were unable to detect the XUT by northern blotting, presumably because it is rapidly degraded by RNA surveillance machineries (using oligo 3; Figure 7A; data not shown). However, quantitative RT-PCR (qRT-PCR) results showed a staggering approximately 25-fold increase in XUT levels in the absence of Nab3 (Figure 7F), clearly demonstrating a role for Nab3 in suppressing the expression of this XUT. The Pol II PAR-CLIP data revealed transcription downstream of the *IPP1* polyadenylation signals (Figure 7A), indicating that a fraction of polymerases did not terminate at these sites. Depletion of Nab3 resulted in an approximately six-fold increase in transcription downstream of the annotated *IPP1* polyadenylation sites (Figure 7G) and low levels of *IPP1* read-through transcripts could be detected by northern blotting and end-point RT-PCR (Figure 7D,H). We conclude that here Nab3 functions as a ‘fail-safe’ terminator by preventing the polymerase from transcribing beyond the *IPP1* polyadenylation sites into the *HHT1* gene. Consistent with the low level of Nrd1 cross-linking in this region, Nrd1 depletion only modestly increased the XUT levels and no significant increase in read-through transcription of *IPP1* could be detected (Figure 7A,D,G). These data indicate a role for Nab3 in fail-safe termination of *IPP1* and suppressing XUT expression, which may interfere with transcription of genes on the opposite strand.

### Nrd1-Nab3-dependent transcription termination of long mRNA transcripts

The level of serine 5 phosphorylated CTD gradually decreases during transcription of coding sequences, and it has been shown that Nrd1-dependent transcription termination becomes less efficient once approximately 900 nucleotides have been transcribed [27,28]. Almost half of the transcripts bound by both Nrd1 and Nab3 in the 3′ UTR were longer than approximately 800 nucleotides (Figure 8A). However, compared to the length distribution of all the analyzed protein coding genes, both proteins did preferentially cross-link to transcripts smaller than 1 kb (Figure 8B). To determine whether Nrd1-Nab3 can terminate transcripts longer than 1 kb, we monitored transcription of the approximately 4.7 kb *YTA7* gene in Nrd1-Nab3 depleted cells. The *YTA7* transcript was selected because significant cross-linking of Nrd1 and Nab3 was detected mainly in the 3′ UTR. Notably, contrary to the *IPP1* transcript, Nrd1-Nab3 cross-linked primarily upstream of polyadenylation sites, indicating that Nrd1-Nab3 termination could precede CPF-dependent termination (Figure 8C,D). The strength of Nrd1-Nab3-dependent transcription termination depends on at least three factors: (1) the number of clustered Nrd1-Nab3 motifs in a sequence, (2) the organization of the binding sites and (3) the presence of AU-rich sequences surrounding the binding sites [16,35]. Three Nab3 motifs were located within 70 nucleotides of the cross-linked Nrd1 motif in the 3′ UTR of *YTA7*, which were surrounded by AU-rich polyadenylation sequences (Figure 8D). This indicates that this region has the required signals for Nrd1-Nab3-directed transcription termination. To address this, we performed qRT-PCR with oligonucleotides that amplify sequences downstream of the *YTA7* 3′ UTR. We also measured *YTA7* mRNA levels by using oligonucleotides that amplify a fragment of the *YTA7* exon (Figure 8E). The results show that depletion of Nrd1 and/or Nab3 led to an increase in transcription downstream of the *YTA7* 3′ UTR (Figure 8E), indicating read through. However, we can not exclude the possibility that these transcripts represent different isoforms of the same gene [48]. As with *IPP1,* depletion of Nab3 had by far the strongest effect (Figure 8E). Strikingly, we could also detect two- to four-fold increase in *YTA7* mRNA levels in the absence of these proteins. This suggests that, by default, a significant fraction of *YTA7* is degraded via the Nrd1-Nab3 termination pathway.

Genome-wide ChIP data had indicated that Nrd1 binding correlated with serine 7 phosphorylation of the Pol II CTD, whereas recruitment of factors required for conventional CPF pathway correlated with serine 2 phosphorylation [37]. Both serine 7 and serine 2 phosphorylation peaked in the 3′ UTR of *YTA7* (Figure 8C) [37], indicating that both the Nrd1-Nab3 and CPF termination pathways are active in this region. This organization of termination signals is frequently found in cryptic transcripts (CUTs) [35], many of which are downregulated via the Nrd1-Nab3 pathway. It appears that a similar mechanism is used to regulate *YTA7* mRNA levels and our bioinformatics analyses suggest that several hundred genes could be regulated in this way; we are currently investigating this in more detail. Transcriptome-wide, the Nrd1-Nab3 UV cross-linking profiles change when cells are starved of glucose [6]. It is conceivable, therefore, that the expression levels of these genes are dictated by the nutrient availability.

## Conclusions

We have presented a comprehensive analysis of Nrd1 and Nab3 PAR-CLIP datasets using the pyCRAC tool suite. We have uncovered more than a thousand potential Nrd1-Nab3 mRNA targets and our data indicate that Nrd1-Nab3 play an important role in the nutrient response and mitochondrial function. We have also provided valuable biological insights into regulation of mRNA transcription by the Nrd1-Nab3 termination pathway. Our data support a role for Nab3 in ‘fail-safe’ termination and regulation of XUT expression. Moreover, we demonstrate that Nrd1-Nab3 can terminate transcription of long transcripts and downregulate mRNA levels by binding to 3′ UTRs. We speculate that at least several hundreds of genes are regulated in this way. We are confident that the analyses presented here will be a useful resource for groups working on transcription termination.

## Materials and methods

### pyCRAC software

The data described here were generated using pyCRAC version 1.1, which can be downloaded from [34]. The Galaxy version is available on the Galaxy tool-shed at [49] and requires pyCRAC to be installed in the /usr/local/bin/ directory.

### Sequence and feature files

All Gene Transfer Format (GTF) annotation and genomic sequence files were obtained from ENSEMBL. Genomic coordinates for annotated CUTs, SUTs, TSSs, polyadenylation sites and UTRs were obtained from the *Saccharomyces* Genome Database (SGD) [22,38-41]. To visualize the data in the UCSC genome browser the pyGTF2bed and pyGTF2bedGraph tools were used to convert pyCRAC GTF output files to a UCSC compatible bed format.

### Raw data processing and reference sequence alignment

Nrd1, Nab3 and Pol II (Rpb2) PAR-CLIP datasets were downloaded from the Gene Expression Omnibus (GEO) database (GSM791764, Nrd1; GDM791765, Rpb2; GSM791767; Nab3). The fastx_toolkit [50] was used to remove low quality reads, read artifacts and adapter sequences from fastq files. Duplicate reads were removed using the pyCRAC pyFastqDuplicateRemover tool. Reads were mapped to the 2008 *S. cerevisiae* genome (version EF2.59) using novoalign version 2.07 [51] and only cDNAs that mapped to a single genomic location were considered.

### Counting overlap with genomic features

PyReadCounters was used to calculate overlap between aligned cDNAs and yeast genomic features. To simplify the analyses, we excluded intron-containing mRNAs. UTR coordinates were obtained from the *Saccharomyces Genome* Database (SGD) [40,52]. The yeast genome version EF2.59 genomic feature file (2008; ENSEMBL) was used for all the analyses described here.

### Calculation of motif false discovery rates

The pyCalculateFDRs script uses a modified version of a FDR algorithm implemented in Pyicos [9]. For a detailed explanation of how the algorithm works, please see the pyCRAC documentation. Reads overlapping a gene or genomic feature were randomly distributed a hundred times over the gene sequence and FDRs were calculated by dividing the probability of finding a region in the PAR-CLIP data with the same coverage by the probability of finding the same coverage in the gene in the randomized data. We only selected regions with an FDR ≤0.01.

### Motif analyses

The motif analyses were performed using the pyMotif tool from the pyCRAC suite. To indicate overrepresentation of a k-mer sequence in the experimental data, pyMotif calculates Z-scores for each k-mer, defined as the number of standard deviations by which an actual k-mer count minus the k-mer count from random data exceeds zero. K-mers were extracted from contigs that mapped sense or anti-sense to yeast genomic features. Repetitive sequences in reads or clusters were only counted once to remove biases towards homopolymeric sequences. Bedtools was used to extract motifs that overlap with genomic features such as exons and UTRs and plots were generated using Gnuplot. The EMBOSS tool fuzznuc was used to extract genomic coordinates for all possible Nrd1 and Nab3 binding and the output files were converted to the GTF format.

### Generation of genome-wide coverage plots

PyBinCollector was used to generate the coverage plots. To normalize the gene lengths, the tool divided the gene sequences over an equal number of bins. For each read, cluster (and their mutations), it calculated the number of nucleotides that map to each bin (referred to as nucleotide densities). To plot the distribution of T-C mutations over the 4 nucleotide Nrd1-Nab3 RNA binding motifs, we added 50 nucleotides up- and downstream of genomic coordinates for each identified motif, and divided these into 104 bins, yielding one nucleotide per bin and the motif start at bin 51. We then calculated the number of T-C substitutions that map to each bin and divided the number by the total number of Ts in each bin, yielding T-C substitution percentages. To plot the distribution of cross-linked motifs around TSSs, we included 500 nucleotides up- and downstream of the start sites and divided these into 1,001 bins, yielding one nucleotide per bin. To generate the heat maps shown in Figures 3 and 4, we used the --outputall flag in pyBinCollector. The resulting data were K-means clustered using Cluster 3.0 [53]. Heat maps were generated using TreeView [54].

### Western and northern blot analyses

Western blot analyses and genetic depletion of Nrd1-Nab3 using *GAL::3HA* strains were performed as previously described [24]. Briefly, cells were grown in YPGalRaf (2% galactose, 2% raffinose) to an OD600 of approximately 0.5 and shifted to YPD medium (2% glucose) for 9 (*GAL::3HA-nrd1/GAL::3HA-nab3*), 10 (*GAL::3HA-nrd1*) or 12 hours (*GAL::3HA-nab3*). Total RNA extraction was performed as previously described [55]. Northern blotting analyses were performed using ULTRAhyb-Oligo according to the manufacturer’s procedures (Ambion Austin, TX, USA). Oligonucleotides used in this study are listed in Table S3 in Additional file 1. Nrd1 and Nab3 proteins were detected using horse radish-conjugated anti-HA antibodies (Santa Cruz, Dallas, TX, USA; 1:5,000)

### RT-PCR analyses

The oligonucleotide primers used for the RT-PCR analyses are listed in Table S3 in Additional file 1. Total RNA was treated with DNase I (Ambion) according to the manufacturer’s instructions. For the qRT-PCR analyses, RNA was reverse-transcribed and amplified using qScript One-Step SYBR Green qRT-PCR (Quanta Bioscience, Gaithersburg, MD, USA), performed on a Roche LightCycler 480 according to the manufacturer’s instructions (Roche, Burgess Hill, UK). Each reaction contained 50 ng template RNA and 250 nM gene-specific primers. Thermal cycling conditions were composed of 50°C for 5 minutes, 95°C for 2 minutes, followed by 40 cycles of 95°C for 3 s, 60°C for 30 s. Appropriate no-RT and no-template controls were included in each assay, and a dissociation analysis was performed to test assay specificity. Relative quantification in gene expression was calculated using the Roche LightCycler 480 Software. YTA7 levels were normalized to the levels of the *PPM2* transcript (NM_00118395) where no significant cross-linking of Nrd1 and Nab3 was detected. For the end-point RT-PCR reactions, 100 ng of total RNA was reverse transcribed using Superscript III at 50°C according to the manufacturers instructions (Invitrogen, Paisley, UK) and 2 μM of *IPP1* reverse primer. The PCR included 200 nM of forward primers. Thermal cycling conditions were 35 cycles of: 95°C for 30 s, 60°C for 30 s and then 72°C for 1 minute.

## Abbreviations

ChIP: Chromatin immunoprecipitation; CLIP: Cross-linking and immunoprecipitation; CPF: Cleavage and polyadenylation; CRAC: Cross-linking and cDNA analysis; CTD: Carboxy-terminal domain; CUT: Cryptic unstable transcript; FDR: False discovery rate; GTF: Gene Transfer Format; PCR: Polymerase chain reaction; Pol: RNA polymerase; qRT-PCR: Quantitative RT-PCR; snoRNA: Small nucleolar RNA; snRNA: Small nuclear RNA; SUT: Stable unannotated transcript; TSS: Transcription start site; UTR: Untranslated region; XUT: Xrn1-sensitive unstable transcript.

## Competing interest

The authors declare that they have no competing interests.

## Authors’ contributions

SW implemented pyCRAC in Galaxy, RDH performed the qRT-PCR experiments, GK aided in the development pyBarcodeFilter and pyMotif. SG conceived and performed the experimental and computational procedures and developed the pyCRAC tools. SW, RDH, GK and SG wrote the paper. All authors read and approved the final manuscript for publication.

## Supplementary Material

Additional file 1: Table S1 PyMotif identified previously described Nrd1-Nab3 RNA binding motifs. Shown are overrepresented 4- to 6-mers (k-mers) and corresponding Z-scores that were isolated from the Nrd1 (11,964) and Nab3 (18,222) intervals with an FDR value of <0.01. Previously identified Nrd1 and Nab3 motifs are highlighted in red and blue, respectively. **Table S2.** Transcripts that contained cross-linked Nrd1-Nab3 motifs in 3′ UTRs. **Table S3.** Oligonucleotides used in this study. Click here for file

Additional file 2: Figure S1 Mutations are enriched in and around Nrd1 and Nab3 RNA binding motifs. **(A, B)** Analysis of T-C mutations found in the PAR-CLIP data near Nab3 and Nrd1 RNA binding motifs. **(C)** Analysis of deletions found in the Nab3 CRAC data around the Nab3 motifs [24]. pyBinCollector was used to calculate the coverage of T-C mutations or deletions in read contigs (see main text) within a 50-nucleotide window over Nab3 (CUUG, UCUU) **(B, C)** and Nrd1 (UGUA, GUAG) **(A)** motifs identified in the genome. To calculate T-C conversion percentages, the number of T-C substitutions was divided by the total number of Ts at each position. The asterisks indicate the positions in the motif where most frequently T-C substitutions were found in the Nrd1-Nab3 motifs. Click here for file
