# Supplementary material for: PAR-CLIP data indicate that Nrd1-Nab3-dependent transcription termination regulates expression of hundreds of protein coding genes in yeast
Source: Genome Biol. 2014 Jan 7;15(1):R8. doi: 10.1186/gb-2014-15-1-r8 (PMC4053934; doi:10.1186/gb-2014-15-1-r8)
Supplement: Additional file 1: Table S1 — PyMotif identified previously described Nrd1-Nab3 RNA binding motifs. Shown are overrepresented 4- to 6-mers (k-mers) and corresponding Z-scores that were isolated from the Nrd1 (11,964) and Nab3 (18,222) intervals with an FDR value of <0.01. Previously identified Nrd1 and Nab3 motifs are highlighted in red and blue, respectively. Table S2. Transcripts that contained cross-linked Nrd1-Nab3 motifs in 3′ UTRs. Table S3. Oligonucleotides used in this study. [file gb-2014-15-1-r8-S1.pdf]

Table S1. PyMotif identified previously described Nrd1-Nab3 RNA binding motifs. Shown are overrepresented 4-6 mers (k-mers) and corresponding Z-scores that were isolated from the Nrd1 (11964) and Nab3 (18222) intervals with an FDR value of < 0.01. Previously identified Nrd1 and Nab3 motifs are highlighted in red and blue, respectively.

Nrd1 PAR-CLIP
11964 significant intervals

k-mer	Z-score
**TGTA**	65.25
**TGTAG**	55.8
**GTAG**	52.35
TTGTA	42.36
CTGTA	37.88
**TGTAA**	37.26
TTGTAG	34.7
TGTAGA	34.22
TTGT	33.37
GTAGA	32.94
CTGT	32.67
ATGTA	31.72
GTAGT	30.54
TGTAGT	29.8
CTGTAG	28.63
GTAA	27.46
ATGTAG	27.08
**TGTAAA**	26
GTGTA	25.3
ATTGTA	24.54
TTTGTA	24.02
TTGTAA	23.46
TCTGTA	22.95
ACTGTA	22.56
ATGT	22.56
TGTAGAA	22.36
CTGTAA	21.86
CTTGTA	21.86
TCTGT	21.74

Nab3 PAR-CLIP
18222 significant intervals

k-mer	Z-score
GTAG	31.73
TGTAG	29.3
CTGT	25.22
TCTG	23.21
TGTA	23.19
GGAC	22.99
CTGTA	22.91
GTAGA	21.78
ACTG	20.1
GACT	19.47
GAAC	19.1
TGTAGA	19.03
TCGG	18.96
CTGTAG	18.96
CTCT	18.95
TTGTAG	18.86
**CTTG**	18.76
ACTGT	18.1
GGACT	17.54
GAACT	17.45
ACTCT	17.28
TGGAC	16.97
GACTG	16.9
**TCTTG**	16.86
CTCTG	16.53
**CTTGT**	16.39
GTAGAA	15.92
ACTGTA	15.8
TCTGT	15.58

Table S2. List of transcripts that contained cross-linked Nrd1-Nab3 motifs in 3' UTRs.

ABF1	CIS1	GCV2	KRE29	NHP2	RAD23	SMF1	TRM5
ABF2	CIS3	GDT1	KSS1	NOP1	RAS2	SMX3	TRM9
ACC1	CKA2	GGA2	LAG1	NOP12	RBG2	SNA3	TRP1
ADE6	CLB1	GIM3	LAP3	NOP14	RDI1	SNF11	TUL1
ADH6	CLF1	GLR1	LAS1	NUC1	REG1	SNF12	UPC2
AIM31	COA2	GLY1	LCB3	NUP100	REX2	SNX3	URA1
AIM4	COG4	GSH2	LIA1	NUP53	RGA1	SOD1	USA1
AIR2	COS8	GSY2	LOT6	OSH6	RGT2	SPB4	UTP23
ALR1	COX16	HAA1	LSM6	OXA1	RMD6	SPC1	VHS1
ALR2	COX17	HAC1	LSP1	OYE2	RNR1	SPI1	VPS4
APA2	COX9	HAL9	LTV1	PAB1	ROT1	SPR6	VPS51
APC11	CSE1	HAP5	LYP1	PAC11	RPA14	SPT7	VPS63
APE3	CYC2	HCH1	LYS14	PAC2	RPC25	SRP14	VTC1
APL2	DAS2	HEM1	MAP2	PAF1	RPC40	SRP40	WRS1
ARG1	DBP6	HIR3	MBA1	PAM18	RPL12A	SSA1	WSS1
ARG8	DED1	HIS7	MCX1	PAN1	RPL41A	SSA2	YAP7
ART10	DIA1	HMF1	MDJ1	PAN2	RPL8A	SSQ1	YBR085C-A
ASF2	DLS1	HMG2	MDN1	PAN6	RPP2B	SST2	YBR126W-B
ASI2	DPB11	HMS2	MDS3	PAT1	RPS5	SSU72	YBR137W
ATP14	DPH5	HMT1	MFA2	PBI2	RRG10	SSY5	YCL041C
ATP16	DSE2	HOR7	MIR1	PBP2	RRG9	STR2	YCL046W
ATP17	ECL1	HPA3	MLH1	PCA1	RRN5	SUR2	YCP4
ATP3	EDC2	HSP12	MLH3U	PCL9	RRP1	TAF9	YCR016W
BBP1	EFT2	HTA1	MNP1	PEP3	RRP8	TAT2	YCR087C-A
BCH2	EGT2	HUA1	MRF1	PEX21	RSM25	TFA1	YDL086W
BEM3	ELC1	HYP2	MRP13	PEX22	RSM7	THP1	YDL094C
BMH1	EMP24	IAH1	MRP21	PHD1	RTC2	THR1	YDL177C
BNA1	ERG11	IBD2	MRPL22	PHO84	RTC6	TIF35	YDL183C
BRO1	ESC2	IES6	MRPL3	PIR1	RTR2	TIM12	YDR157W
BSC2	ESS1	ILV5	MRPL38	PIS1	SAM1	TIM54	YDR239C
BUB3	FAR3	IMH1	MRPL4	PKH1	SAM2	TMA19	YEA6
BUD26	FCJ1	IML3	MSC3	PKP1	SAM3	TMA22	YEL045C
CAB2	FEN1	IRA1	MSL5	POC4	SAM35	TOF2	YEL057C
CAD1	FMP16	IRC21	MSN1	POL30	SBE22	TOM40	YER064C
CBK1	FRE4	IRC7	MUQ1	PSA1	SDS3	TOS1	YER088W-B
CCA1	FUI1	ISU1	NAB2	PSD2	SEC53	TPK3	YER140W
CCE1	FYV12	ITC1	NAB3	PTI1	SFK1	TPS2	YFR020W
CDC8	GAS1	JIP4	NAM8	PUS9	SHO1	TRA1	YGL088W
CHA4	GCR2	JLP2	NAM9	QDR3	SIP5	TRE1	YGL108C
CHC1	GCS1	KGD2	NCL1	RAD10	SKY1	TRI1	YGR021W

Table S2 continued.

YGR025W	YOR248W
YGR050C	YOR316C-A
YGR069W	YPL066W
YGR079W	YPL107W
YGR130C	YPL191C
YGR240C-A	YPL229W
YHL042W	YPR097W
YHR049C-A	YPR126C
YIL096C	YPR160C-A
YIL102C-A	YRO2
YJR107W	YSP1
YJR124C	YTA12
YKL047W	YTA7
YKL061W	ZEO1
YKL068W-A	
YKL100C	
YLF2	
YLH47	
YLR108C	
YLR143W	
YLR171W	
YLR286W-A	
YLR287C	
YLR419W	
YME1	
YMR013W-A	
YMR103C	
YMR122W-A	
YMR187C	
YNL010W	
YNL024C	
YNL097C-B	
YNL140C	
YNL162W-A	
YNR021W	
YNR040W	
YOL013W-A	
YOL097W-A	
YOR060C	
YOR097C	

Table S3. Oligonucleotides used in this study

Oligonucleotides used for quantitative RT-PCR:

Gene	Forward primer (5'-3')	Reverse primer (5'-3')
HHT1_3UTR	CAGGCCTTGTAGGAGGCAAGATTT	CCACGTATGCGGCTTCAAGTTGTT
HHT1_exon	CAGGTACTGTTGCTTTGAGAGA	CACCGATGGCAGAAGATTGA
YTA7_3UTR	TCTGAATAAGCAGGAGTCCATAATGT	ACCCGAAACGTATGCCAGAACAGT
YTA7_exon	GCAAATTCAGACCAGGAAATGG	AGTGCATAGGGACCTCAGAT
PPM2	ATCCGCTGAGTGAGACGCTAACAT	TGGCACCGCCTCCAATGATATGTA
IPP1_exon	CGTAAGCCACCCAGAAACTAA	CGGTCTCACCTTCATCCAATAA
IPP1_3UTR	CGAGGGTATCGCCAGGA	GGTTTACTGGGTTCTTTAACAGTTG

Oligonucleotides used for end-point RT-PCR:

Gene	Forward primer (5'-3')	Reverse primer (5'-3')
IPP1_exon	CTGGTAAGTCTTCTGACAGCAAG	GGAGATGAAGAACCACTTGTC
IPP1_downstream	CTGGTAAGTCTTCTGACAGCAAG	GATTTGTTTTTCCTGGCGATACC

Oligonucleotides used for Northern Blotting:

Gene	Sequence (5'-3')
HHT1_exon	GGCGTGAATGGCAGCCAAGTTGGTATCTTCAAATAAAGAGACTAAGTAGGC TTCGACAGATTCTTGCAAGGCACCG
HHT1_downstream	CCACATGGAAAGCCATAAATCTTGCCTCCTACAAGGCCTGGC
IPP1_exon	GGGAAAGCACCATAGTTGTGAATGTAACCATGGTGAGGGAAACAG
IPP1_downstream	GCCAGGCCTTGTAGGAGGCAAGATTTATGGCTTTCCATGTGG
snR13	GCCAAACAGCAACTCGAGCCAAATGCACTCATATTCATC
U2_snRNA	CCCTTTAGCAAACCTCCATTTATATCCTGCGAGAAGAGCTCCTTC
