# Supplementary figures and images for: PAR-CLIP data indicate that Nrd1-Nab3-dependent transcription termination regulates expression of hundreds of protein coding genes in yeast
Source: Genome Biol. 2014 Jan 7;15(1):R8. doi: 10.1186/gb-2014-15-1-r8 (PMC4053934; doi:10.1186/gb-2014-15-1-r8)

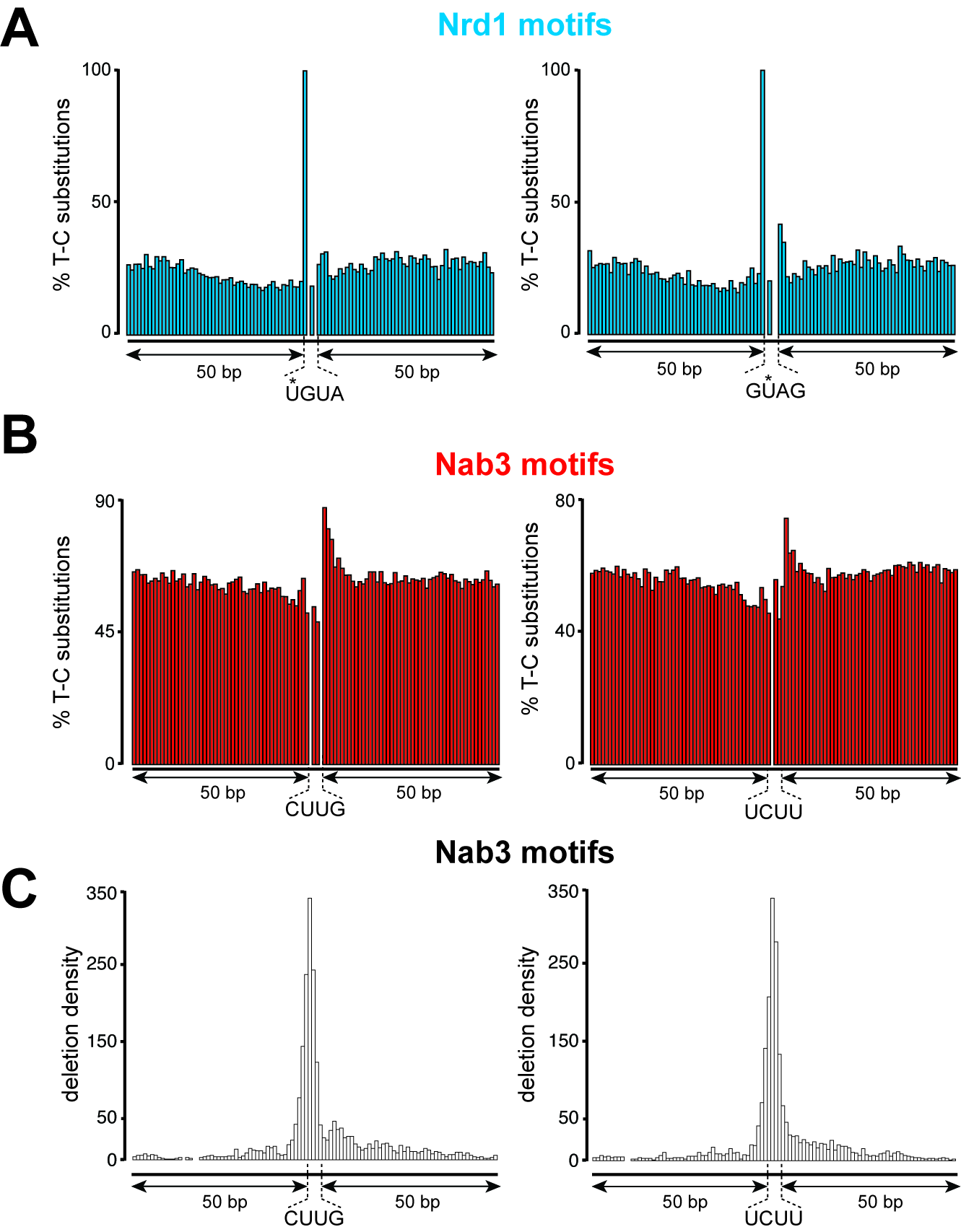

Supplement: Additional file 2: Figure S1 — Mutations are enriched in and around Nrd1 and Nab3 RNA binding motifs. (A, B) Analysis of T-C mutations found in the PAR-CLIP data near Nab3 and Nrd1 RNA binding motifs. (C) Analysis of deletions found in the Nab3 CRAC data around the Nab3 motifs [24]. pyBinCollector was used to calculate the coverage of T-C mutations or deletions in read contigs (see main text) within a 50-nucleotide window over Nab3 (CUUG, UCUU) (B, C) and Nrd1 (UGUA, GUAG) (A) motifs identified in the genome. To calculate T-C conversion percentages, the number of T-C substitutions was divided by the total number of Ts at each position. The asterisks indicate the positions in the motif where most frequently T-C substitutions were found in the Nrd1-Nab3 motifs. [file gb-2014-15-1-r8-S2.tiff]
